# Alkylation of nucleobases by 2-chloro-*N,N*-diethylethanamine hydrochloride (CDEAH) sensitizes *PARP1*-deficient tumors

**DOI:** 10.1093/narcan/zcad042

**Published:** 2023-08-07

**Authors:** Minwoo Wie, Keon Woo Khim, Arnold S Groehler IV, Soomin Heo, Junhyeok Woo, Kook Son, Eun A Lee, Jae Sun Ra, Sung You Hong, Orlando D Schärer, Jang Hyun Choi, Kyungjae Myung

**Affiliations:** Center for Genomic Integrity, Institute for Basic Science, Ulsan 44919, Republic of Korea; Department of Biological Sciences, Ulsan National Institute of Science and Technology, Ulsan 44919, Republic of Korea; Center for Genomic Integrity, Institute for Basic Science, Ulsan 44919, Republic of Korea; Department of Biological Sciences, Ulsan National Institute of Science and Technology, Ulsan 44919, Republic of Korea; Center for Genomic Integrity, Institute for Basic Science, Ulsan 44919, Republic of Korea; Center for Genomic Integrity, Institute for Basic Science, Ulsan 44919, Republic of Korea; Department of Biomedical Engineering, Ulsan National Institute of Science and Technology, Ulsan 44919, Republic of Korea; Department of Chemistry, Ulsan National Institute of Science and Technology, Ulsan 44919, Republic of Korea; Center for Genomic Integrity, Institute for Basic Science, Ulsan 44919, Republic of Korea; Center for Genomic Integrity, Institute for Basic Science, Ulsan 44919, Republic of Korea; Center for Genomic Integrity, Institute for Basic Science, Ulsan 44919, Republic of Korea; Department of Chemistry, Ulsan National Institute of Science and Technology, Ulsan 44919, Republic of Korea; Center for Genomic Integrity, Institute for Basic Science, Ulsan 44919, Republic of Korea; Department of Biological Sciences, Ulsan National Institute of Science and Technology, Ulsan 44919, Republic of Korea; Center for Genomic Integrity, Institute for Basic Science, Ulsan 44919, Republic of Korea; Department of Biological Sciences, Ulsan National Institute of Science and Technology, Ulsan 44919, Republic of Korea; Center for Genomic Integrity, Institute for Basic Science, Ulsan 44919, Republic of Korea; Department of Biomedical Engineering, Ulsan National Institute of Science and Technology, Ulsan 44919, Republic of Korea

## Abstract

Targeting *BRCA1*- and *BRCA2*-deficient tumors through synthetic lethality using poly(ADP-ribose) polymerase inhibitors (PARPi) has emerged as a successful strategy for cancer therapy. PARPi monotherapy has shown excellent efficacy and safety profiles in clinical practice but is limited by the need for tumor genome mutations in *BRCA* or other homologous recombination genes as well as the rapid emergence of resistance. In this study, we identified 2-chloro-*N,N*-diethylethanamine hydrochloride (CDEAH) as a small molecule that selectively kills *PARP1*- and xeroderma pigmentosum A-deficient cells. CDEAH is a monofunctional alkylating agent that preferentially alkylates guanine nucleobases, forming DNA adducts that can be removed from DNA by either a PARP1-dependent base excision repair or nucleotide excision repair. Treatment of *PARP1*-deficient cells leads to the formation of strand breaks, an accumulation of cells in S phase and activation of the DNA damage response. Furthermore, CDEAH selectively inhibits *PARP1*-deficient xenograft tumor growth compared to isogenic *PARP1*-proficient tumors. Collectively, we report the discovery of an alkylating agent inducing DNA damage that requires PARP1 activity for repair and acts synergistically with PARPi.

## Graphical Abstract

**Graphical Abstract:**
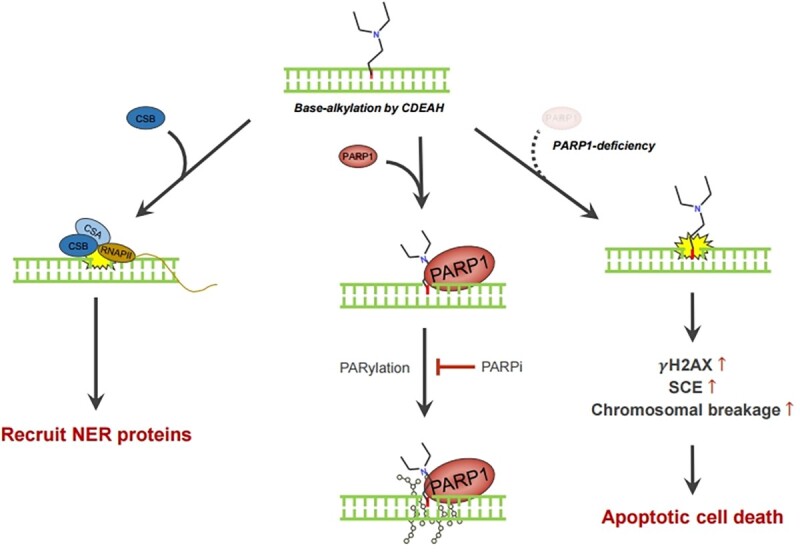

## INTRODUCTION

Poly(ADP-ribose) polymerase 1 (PARP1) promotes DNA repair by binding to DNA breaks and by attaching ADP-ribose polymers to itself and a number of other proteins to regulate DNA repair. PARP1 has been shown to have a role in many DNA pathways and has a special role in base excision repair (BER) (1,2). In addition, PARP1 has been shown to have a role in regulating replication fork speed (3,4), protecting stalled replication forks (5,6) and preventing the formation of gaps formed on the lagging strand by incomplete Okazaki fragment synthesis in lagging strand DNA synthesis (3,5–8). Therefore, PARP1 dysfunction or inhibition in homologous recombination (HR)-deficient cells leads to the accumulation of replication gaps in S phase, and the exposed lagging strand gaps become toxic to cells (7). Thus, inhibiting PARP1 is synthetic lethal to cells with defects in HR genes (9,10). Such synthetic lethality, referring to the cell-lethal effects upon the inactivation of two genetically distinct pathways, is a useful approach to selectively kill cells with defects in a DNA repair pathway (11–13). In particular, synthetic lethality provides a conceptual framework for the development of drugs that are selectively toxic in specific genetic backgrounds associated with tumors. In addition to PARP1 inhibition, prominent recent examples are the use of PolQ inhibitors to inhibit alternative end joining pathways to target tumors carrying a mutation in the *BRCA1* or *BRCA2* (14,15) or inhibition of the Werner protein in tumors with mismatch repair (MMR) deficiencies (16).

Currently, certain types of breast (e.g., high-grade serous ovarian cancer or triple-negative breast cancer), ovarian, pancreatic or prostate cancers carrying a *BRCA1* or *BRCA2* mutation can be treated with PARP inhibitors (PARPi) as a first-line treatment (17). Although PARPi monotherapy has shown promising efficacy and safety profiles in clinical practice, its major limitations are the need for specific alterations in HR genes in tumors and the rapid emergence of resistance (18–20). Many tumors that initially respond to PARPi treatment eventually recur through compensatory mutations that restore HR activity or stimulate the activity of alternative repair pathways (11). To overcome these issues in clinical practice, various combinatorial treatments of PARPi with drugs targeting other pathways are currently being tested (11,17).

ATAD5 (ATPase family AAA domain-containing protein 5) is a human protein encoded by the *ATAD5* gene that belongs to the AAA+ ATPase family. Its main function is to ensure DNA replication and maintain genomic stability by regulating DNA replication initiation and elongation, responding to DNA damage and stabilizing stalled replication forks (21–28). ATAD5 dysregulation or mutations have been linked to various types of cancer (29–31). ATAD5 is also a useful biomarker for detecting genotoxic compounds, as its protein levels increase after DNA damage (32). To identify small molecules eliciting DNA replication stresses, a HEK293T cell line stably expressing the luciferase-tagged ATAD5 (ATAD5-luc cell), which measures the luciferase activity as a readout to measure the level of ATAD5 expression in response to DNA damage or replication stress (33), was used for the identification of small molecules inducing DNA replication stresses. We reasoned that through a screen for small molecules that cause replication stress combined with an analysis of the pathway(s) inhibited by any of the hits, we would be likely to identify molecules that act synergistically with PARPi. Using this assay, we screened a 344,385 small molecule library (the National Institute of Health’s Molecular Libraries Probe Production Centers Network) and identified 289 small molecules that activated expression of the ATAD5 reporter gene. Among the positive hits, we have already characterized the small molecule baicalein, which can selectively kill MMR-deficient tumors through its preferential interaction with mismatched DNA and the MSH2–MSH6 complex for activation of the ATM–CHK2 pathway (34).

To begin to identify the molecular mechanisms by which other identified small molecules cause DNA replication stress and DNA damage, we treated various cell lines with mutations in DNA repair genes to identify whether any of them caused synthetic lethality. In this screen, we identified 2-chloro-*N*,*N*-diethylethanamine hydrochloride (CDEAH), monofunctional or half-nitrogen mustard, as a small molecule that selectively kills *PARP1*- and xeroderma pigmentosum A (*XPA*)-deficient cells in cell culture and xenograft models. CDEAH preferentially alkylates guanine residues in DNA, forming adducts that can be removed by either BER or nucleotide excision repair (NER). As the intermediate in BER, an abasic site is bound by PARP. Collectively, we report a potential synergistic treatment option of PARPi by enhancing DNA damage that depends on PARP1-dependent BER mechanisms.

## MATERIALS AND METHODS

### Cell lines

HCT116 (ATCC), HEK293T (ATCC), XP2OS [*XPA* mutated c.390-1G>C (IVS3-1G>C) to create a splicing acceptor in exon 3], XP2OS expressing wild-type (WT) XPA (XPA complemented) (35), U2OS (ATCC) and HEK293T ATAD5-LUC (33) cells were cultured in Dulbecco’s modified Eagle’s medium (Gibco^®^) containing 10% fetal bovine serum (FBS, Merck) and 1% antibiotic–antimycotic [penicillin 10,000 units/ml, streptomycin 10,000 μg/ml and Fungizone^®^ (amphotericin B) 25 μg/ml, Gibco^®^] at 37°C in the presence of 5% CO_2_. HCT116 *PARP1* knockout (KO) cells were kindly gifted by Dr Eric Hendrickson (University of Minnesota Medical School, USA). U2OS *XPA*, *XPC* and *CSB* KO cells were kindly gifted by Dr Martijn S. Luijsterburg (Leiden University Medical Center, the Netherlands) (36). TK6 BRCA2-mAID-GFP cells were kindly gifted by Dr Shunichi Takeda (Kyoto University, Japan) and cultured in RPMI 1640 containing 5% HIDHS (Gibco, New Zealand, #16050, not heat inactivated), 0.2 mg/ml sodium pyruvate (Gibco, 11360-070, 11 g/l) and 1% penicillin/streptomycin mix (Gibco, 15140-122, 100 ml). Indole-3-acetic acid (500 μM), a natural auxin, was added to the culture medium to induce degradation of AID-tagged BRCA2. HAP1 *PARP1*, *XPA*, *53BP1*, *XRCC4* and *RAD52* KO cells were purchased from Horizon. HAP1 (Horizon) cells were cultured in Iscove’s modified Dulbecco’s medium containing 10% FBS and 1% antibiotic–antimycotic at 37°C in the presence of 5% CO_2_.

### Plasmids, chemicals and antibodies

Bromo-*N*,*N*-diethylethanamine hydrobromide, CDEAH, 2,2,2-trifluoroethanol (TFE), 5-fluorouridine (5-FUrd), adenine, bromodeoxyuridine (BrdU), cytosine, guanine, methyl methanesulfonate (MMS), temozolomide (TMZ), sodium acetate and thymidine were purchased from Sigma–Aldrich. Olaparib was purchased from Selleckchem (AZD2281). Colcemid KaryoMAX Solution, 10 μg/ml (cat. 15210-016, 10 ml) and Giemsa stain (cat. 10092-013, 100 ml) were purchased from Gibco BRL. Anti-XPA (sc-853), anti-XPC (sc-74411) and anti-alpha-Tubulin (DM1A, sc-32293) were purchased from Santa Cruz. Anti-γ-H2AX (Ser139, 05-636) was purchased from Merck Millipore. Anti-PARP1 (ALX-210-302-R100) was purchased from Enzo Life Sciences. Anti-CSB (GTX104589) was purchased from GeneTex. Anti-PAR (4335-MC-100) was purchased from R&D Systems.

### ATAD5-luciferase assay

HEK293T ATAD5-LUC cells (33) were plated at a density of 15,000 cells per well in a 96-well white, assay plate (Costar). After 24 h, cells were treated with 5-FUrd or CDEAH and incubated for an additional 24 h. Luciferase activity was measured by adding ONE-Glo luciferase reagent (Promega) to each well and measuring luminescence intensity with a Synergy NEO2 Hybrid Multi-Mode Reader (BioTek).

### Immunoblot analysis

Whole-cell extracts were isolated and immunoblot analysis was performed as previously described (24) with slight modifications. Briefly, whole-cell extracts were isolated by incubating cells on ice with Benzonase^®^ nuclease (250 units/μl, Enzynomics) in RIPA buffer [50 mM Tris–HCl (pH 8.0), 150 mM NaCl, 5 mM EDTA, 1% Triton X-100™, 0.1% sodium dodecyl sulfate, 0.5% sodium deoxycholate, Halt™ Protease & Phosphatase Single-Use Inhibitor Cocktail] for 40 min, followed by sonication and centrifugation. For immunoblot analysis, proteins were resolved by sodium dodecyl sulfate–polyacrylamide gel electrophoresis and transferred to a nitrocellulose membrane. The membrane was incubated for 20 min in Tris-buffered saline containing 0.1% Tween 20 (TBS-T) supplemented with 5% skim milk for blocking, followed by overnight incubation with a primary antibody at 4°C. The blots were washed and incubated with horseradish peroxidase-conjugated secondary antibody (Enzo Life Sciences) in TBS-T at 1:5,000 dilution for 1 h. Signals were detected using an enhanced chemiluminescent reagent (Thermo Fisher Scientific) by an automated imaging system (ChemiDoc™; Bio-Rad Laboratories).

### Flow cytometry

Cells were washed with phosphate-buffered saline (PBS) and fixed with 70% ethanol in PBS overnight. Fixed cells were then washed with PBS and incubated with 0.2 mg/ml RNase A in PBS at 37°C for 1 h. DNA was stained with 10 μg/ml propidium iodide in PBS. Flow cytometry was performed on a FACSVerse™ flow cytometer using BD FACSuite™ software (BD Biosciences). Data analysis was performed using the FlowJo software.

### Analysis of abnormal chromosomes

Cells were incubated with 0.2 μg/ml colcemid for 3 h, and then metaphase cells were harvested by trypsinization. The cells were then swollen in 0.075 M KCl at 37°C for 15 min and fixed with methanol:acetic acid (3:1) twice. Cells were dropped onto glass microscope slides and stained with 5% Giemsa stain. Images were acquired using a fluorescence microscope (BX53; Olympus, Tokyo, Japan). At least 20 metaphase cells were taken randomly from each condition.

### Sister-chromatid exchange assay

Cells were cultured in media containing BrdU at a final concentration of 25 μg/ml for 48 h. CDEAH was added 24 h before harvest, and colcemid (0.2 μg/ml) was added for the final 3 h. Metaphase cells were harvested by trypsinization. The cells were then swollen in 0.075 M KCl at 37°C for 15 min and fixed with methanol:acetic acid (3:1) twice. Cells were dropped onto glass microscope slides and stained with 5% Giemsa stain. Images were acquired using a fluorescence microscope (BX53; Olympus). At least 20 metaphase cells were taken randomly from each condition.

### Apoptosis assay

Apoptotic cell death was quantified using an Annexin V Alexa Fluor™ 488 conjugate (A13201, Thermo Fisher Scientific) and a BD FACSVerse instrument with FlowJo software (version 10) according to the manufacturer’s instructions.

### Comet assay

The comet assay was performed using a CometChip^®^ (Trevigen) according to the manufacturer’s instructions. In brief, single-cell suspensions were prepared in 6 ml medium with 1.0 × 10^5^ cells/ml density. Aliquots of 100 μl cells per well were applied to a CometChip and incubated in a tissue culture incubator for 10 min, with gentle shaking three times in 10 min intervals to spread cells evenly. Medium was removed and each CometChip from the 96-well CometChip^®^ system was gently washed with 5 ml PBS twice. The CometChip was then covered with 6 ml of 1% 45°C low-melting agarose in PBS. After the solidification of the agarose, the slide was immersed in a lysis solution (Trevigen) overnight at 4°C. The CometChip was equilibrated twice in an alkaline solution at 4°C for 20 min, electrophoresed at 4°C for 50 min at 22 V in an alkaline solution, neutralized twice at 4°C for 15 min in fresh 0.4 M Tris (pH 7.4) buffer and then equilibrated at 4°C for 30 min in 20 mM Tris (pH 7.4) buffer. DNA in CometChips was stained with 0.2× SYBR^®^ Gold in 20 mM Tris (pH 7.4) buffer at room temperature for 2 h. Images were acquired with a fluorescence microscope (BX53; Olympus) and the tail moment was calculated using the Comet analysis software (Trevigen).

### Cell viability assay

Cells were plated in white, solid-bottom 96-well plates for a 2-day incubation period. HAP1 and HCT116 cells were plated at a final density of 5,000 cells per well, while XP2OS cells were plated at a density of 3,000 cells per well and incubated for 1 day prior to treatment with the specified compounds. Cell viability was measured 2 days after treatment using Cell Titer-Glo (Promega) according to the manufacturer’s instructions.

For cell viability assay after a 6-day incubation protocol, cells were plated in black, solid-bottom 96-well plates. HAP1 cells were plated at a final density of 600 cells per well, while TK6 cells and HCT116 cells were plated at a final density of 700 cells per well and incubated for 1 day prior to treatment with the indicated compounds. Cell viability was measured 6 days after treatment using Cell Titer-Blue (Promega) according to the manufacturer’s instructions. Viability was quantified in a Synergy NEO2 Hybrid Multi-Mode Reader (BioTek). The Chou–Talalay combination index method (37) was utilized to assess the effects of the drug combination. The analysis was performed using the freely accessible CompuSyn software tool.

### Alkylation of nucleobases with CDEAH

#### Reaction of adenine with CDEAH

**Scheme 1.**
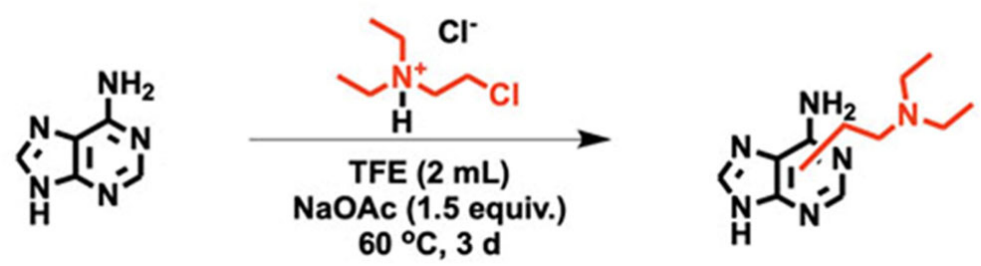
Reaction of adenine with CDEAH.

CDEAH (28.8 mg, 0.2 mmol, 1.0 equiv.) was added to a solution of adenine (27.0 mg, 0.2 mmol, 1.0 equiv.) dissolved in TFE (2.0 ml) (see Scheme 1). Sodium acetate (24.6 mg, 0.3 mmol, 1.5 equiv.) was added to adjust the pH to neutral. The reaction mixture was stirred at 37 or 60°C for 3 days. After incubation, the crude reactant was filtered through a syringe filter to remove precipitates. The filtrated chemicals were characterized by ultra-performance liquid chromatography–high-resolution accurate mass-parallel reaction monitoring (UPLC–HRAM-PRM) as described below.

#### Reaction of guanine with CDEAH

**Scheme 2.**
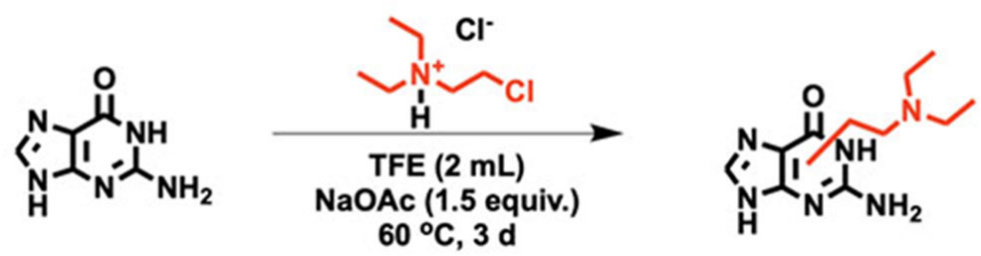
Reaction of guanine with CDEAH.

CDEAH (28.8 mg, 0.2 mmol, 1.0 equiv.) was added to a solution of guanine (30.2 mg, 0.2 mmol, 1.0 equiv.) dissolved in TFE (2.0 ml) (see Scheme 2). Sodium acetate (24.6 mg, 0.3 mmol, 1.5 equiv.) was added to adjust the pH to neutral. The reaction mixture was stirred at 37 or 60°C for 3 days. After incubation, the crude reactant was filtered through a syringe filter to remove precipitates. The filtrated chemicals were characterized by UPLC–HRAM-PRM as described below.

#### Reaction of thymine with CDEAH

**Scheme 3.**
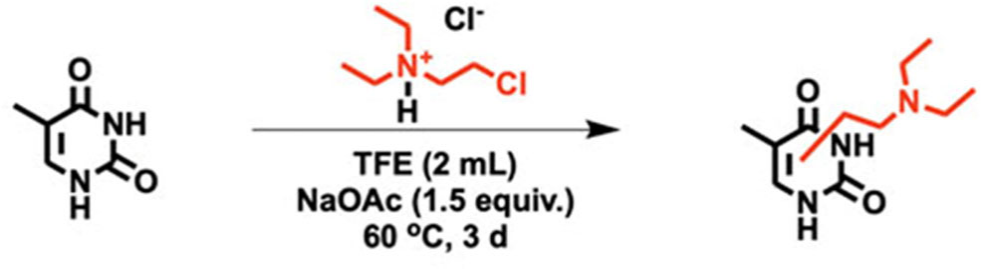
Reaction of thymine with CDEAH.

CDEAH (28.8 mg, 0.2 mmol, 1.0 equiv.) was added to a solution of thymine (25.2 mg, 0.2 mmol, 1.0 equiv.) dissolved in TFE (2.0 ml) (see Scheme 3). Sodium acetate (24.6 mg, 0.3 mmol, 1.5 equiv.) was added to adjust the pH to neutral. The reaction mixture was stirred at 37 or 60°C for 3 days. After incubation, the crude reactant was filtered through a syringe filter to remove precipitates. The filtrated chemicals were characterized by UPLC–HRAM-PRM as described below.

#### Reaction of cytosine with CDEAH

**Scheme 4.**
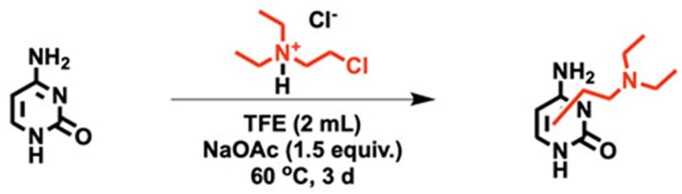
Reactions of cytosine with CDEAH.

CDEAH (28.8 mg, 0.2 mmol, 1.0 equiv.) was added to a solution of cytosine (22.2 mg, 0.2 mmol, 1.0 equiv.) dissolved in TFE (2.0 ml) (see Scheme 4). Sodium acetate (24.6 mg, 0.3 mmol, 1.5 equiv.) was added to adjust the pH to neutral. The reaction mixture was stirred at 37 or 60°C for 3 days. After incubation, the crude reactant was filtered through a syringe filter to remove precipitates. The filtrated chemicals were characterized by UPLC–HRAM-PRM as described below.

### SPE purification of alkylated nucleobases

An aliquot of each alkylated *N-*[diethylamino(ethyl)]-nucleobase (DEAE-nucleobase) reaction was reconstituted in 1 ml of 5% methanol in water and sonicated for 30 min. Following sonication, the solution was centrifuged at 14,000 rcf at room temperature for 10 min to pellet the solid precipitate. Oasis^®^ HLB 30 mg extraction cartridges (Waters, Milford, MA) were placed on a vacuum manifold and conditioned with two additions of 1 ml of water, and then 1 ml of methanol with a gentle vacuum applied. The sample solutions were then loaded onto a column, followed by washing twice with 2 ml of 5% methanol in water. DEAE-purine nucleobases and DEAE-pyrimidine nucleobases were eluted with two additions of 500 μl of 100% methanol. Collected elution was concentrated by centrifugal vacuum and stored at −20°C for future analysis.

### Alkylation of calf thymus DNA with CDEAH

An aliquot of 100 μg of calf thymus DNA (CTDNA) dissolved in water or PBS was diluted to 190 μl with water or PBS, followed by adding 10 μl of 2 mM CDEAH to yield a final concentration of 100 μM. The solutions were then incubated at 37°C for 16 h to allow alkylation of nucleotides, followed by heating at 70°C for 1 h to depurinate DEAE-purine bases. The released DEAE-purine nucleobases were separated from the DNA backbone by centrifugation at 14,000 rcf at 4°C for 10 min through Nanosep^®^ centrifugal devices with Omega™ 10-kDa membranes. The filters were further washed using an equal volume of deionized (DI) water twice, and 100 μl of 50:50 acetonitrile (ACN):DI water once. All collected solutions (depurination solution) were concentrated to dryness by centrifugal vacuum and stored at −20°C for future experiments. The DNA backbone in the filter was resuspended in 100 μl water (DNA backbone solution), recovered from the filter and stored at −20°C for future experiments.

### Characterization of alkyl-nucleobase standards by UPLC–HRAM-PRM

Alkylation of purine and pyrimidine nucleobases by CDEAH was confirmed by analyzing the solid-phase extraction (SPE)-purified reaction products by a UPLC–HRAM-PRM assay in positive mode as follows: a Hypersil GOLD 1.9 μm C18 column (100 mm × 1.0 mm) was operated using a gradient of buffer A (15 mM ammonium acetate, pH 7.0) and buffer B (100% acetonitrile) at 0.05 ml/min starting at 2% buffer B for 2 min, linearly increased to 25% buffer B over 8 min, followed by an increase to 50% buffer B over 20 min, then an increase to 80% buffer B over 2 min, held constant at 80% buffer B for 2 min, followed by a decrease to 2% buffer B over 2 min and finally re-equilibrated at 2% buffer B for 9 min. Mass spectrometry (MS) settings were as follows: electrospray voltage, 3,500 V; capillary temperature, 320°C; full scan AGC, 1 × 10^6^; full scan resolution, 70,000; HESI temperature, 150°C; sheath gas, auxiliary gas and sweep gas flow rates, 35, 10 and 1 arbitrary units, respectively. The PRM MS settings were as follows: PRM AGC, 5 × 10^4^; PRM resolution, 35,000. The DEAE-purine and DEAE-pyrimidine nucleobase PRM settings were as follows: ESI^+^-PRM N7-DEAE-guanine and N9-DEAE-guanine: *m*/*z* (+1) = 251.1615 from 9 to 14.0 min; ESI^+^-PRM N1-DEAE-adenine, N3-DEAE-adenine and N7-DEAE-adenine (possibility of N9-DEAE-adenine): *m*/*z* (+1) = 235.1661 from 12 to 15 min; ESI^+^-PRM N1-DEAE-cytosine and N3-DEAE-cytosine: *m*/*z* (+1) = 211.1550 from 4 to 7 min and from 8 to 13 min; and ESI^+^-PRM N1-DEAE-thymine and N3-DEAE-thymine: *m*/*z* (+1) = 226.1547 from 10 to 13 min.

### Analysis of CTDNA alkylation by CDEAH by UPLC–HRAM-PRM

The depurination solution from above was reconstituted in 50 μl water and measured by a microvolume UV spectrophotometer (Thermo Scientific^™^ NanoDrop) using the extinction coefficient for guanosine to confirm that nucleobases were present. The equivalent of 1.2 μg of CTDNA from the depurination solution was analyzed by the DEAE-purine UPLC–HRAM-PRM method described above.

The DNA backbone solution from above was measured by a microvolume UV spectrophotometer, and a 25 μg aliquot of CTDNA was diluted to 150 μl of 1× NEB nucleoside digestion mix reaction buffer and incubated with 2.5 μl NEB nucleoside digestion mix (1 μl mix per 10 μg CTDNA) at 37°C for 4 h. Following incubation, the digestion enzymes were removed by centrifugation at 14,000 rcf at 4°C for 10 min through Nanosep^®^ centrifugal devices with Omega™ 10-kDa membranes. The filters were further washed using an equal volume of DI water one additional time, and 100 μl of 50:50 ACN:DI water two additional times. All collected solutions were concentrated to dryness by centrifugal vacuum. The resulting digest was reconstituted in 25 μl LC–MS water and a 5 μg aliquot of CTDNA was analyzed by a modified DEAE-pyrimidine nucleoside UPLC–HRAM-PRM assay as described: ESI^+^-PRM DEAE-2′-deoxycytidine: *m*/*z* (+1) = 327.20270 from 11 to 14.0 min and ESI^+^-PRM DEAE-thymidine: *m*/*z* (+1) = 342.20230 from 11 to 14 min. UPLC–PRM MS settings were the same as described for the DEAE-purine base assay described above.

### Mouse xenograft

Animal experiments were performed by following the guideline of UNIST’s Institutional Animal Care and Use Committee. Seven-week-old male BALB/c nude mice were purchased from Orient Bio (Gyeonggi, Republic of Korea). Four million cells of WT or *PARP1* KO HCT116 were suspended in 150 μl of sterile HBSS (Welgene, Gyeongbuk, Republic of Korea) and injected subcutaneously into the left flank. Intratumoral injection of vehicle (PBS) or CDEAH was conducted every 3 days for 16 days after each tumor size reached ∼200 mm^3^. Tumor sizes were measured using calipers every 3 days for 16 days following drug treatment. Tumor volume was measured by the following formula: $V = {( {{\mathrm{width}}} )}^2 \times {\mathrm{\ length }} \times ({1}/{2})$. All mice were euthanized to harvest tumors for immunostaining.

### Immunohistochemistry

Hematoxylin and eosin (H&E) staining, TUNEL and γ-H2AX immunostaining were commercially performed by Histoire (Seoul, Republic of Korea). Collected tumors were fixed in formalin and picked up by Histoire. Detailed immunostaining procedures can be checked by accessing the Histoire’s website (http://www.histoire.co.kr/).

### Statistical analysis

**Figure 1.**
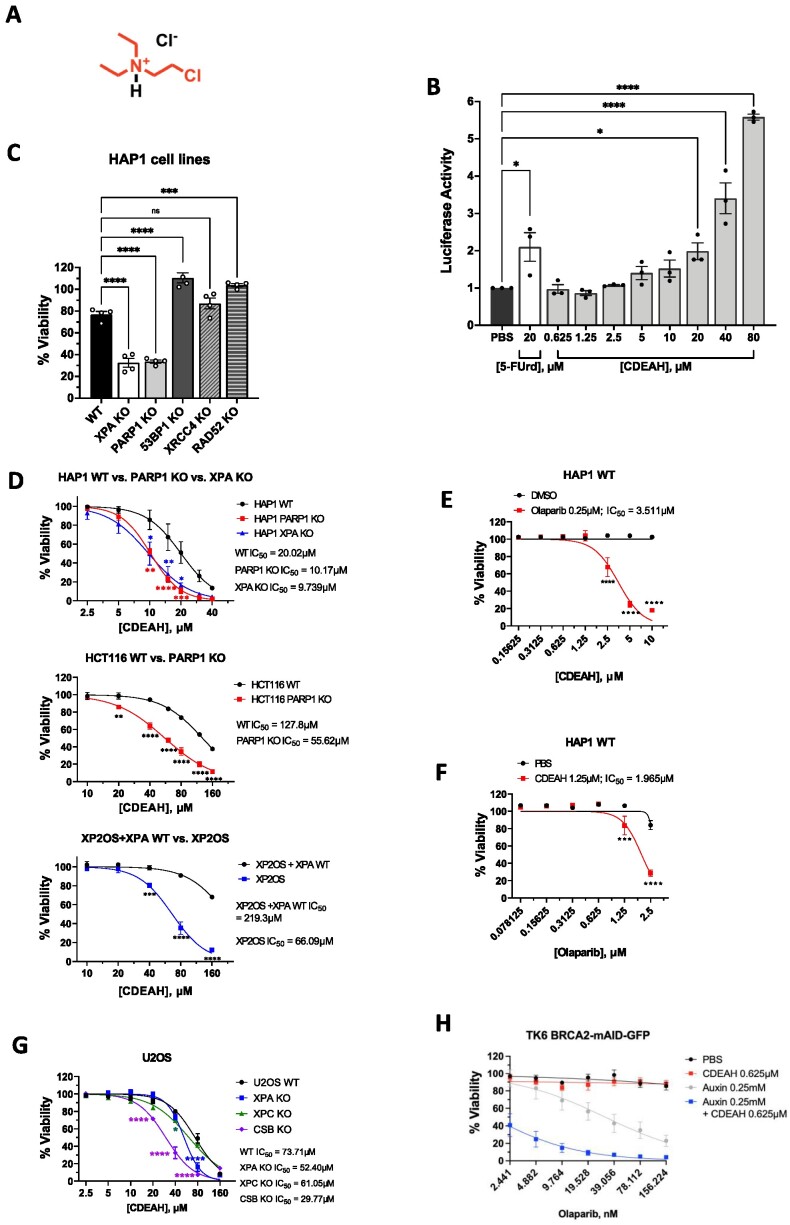
CDEAH selectively kills XPA- and PARP1-deficient cells. (**A**) Structure of CDEAH. (**B**) CDEAH induces DNA replication stress. HEK293T cells stably expressing luciferase-fused ATAD5 were grown in 96-well plates at a density of 15,000 cells per well and treated with 5-FUrd as a positive control or CDEAH for 24 h. The luciferase activity was measured using the ONE-Glo luciferase reagent. (**C**) CDEAH kills XPA- and PARP1-deficient HAP1 cells better than WT. HAP1 cells deficient in XPA, PARP1, 53BP1, XRCC4 and RAD52 or WT were cultured in 96-well plates at a density of 5,000 cells per well and exposed to 20 μM CDEAH for 48 h. Cell viability was determined using Cell Titer-Glo reagent. (**D**) Cell survival response to dose-dependent CDEAH treatment. Indicated cells were grown in 96-well plates and treated with various doses of CDEAH for 48 h. Cell viability was determined using Cell Titer-Glo reagent. (**E**) Cell survival in response to various doses of CDEAH with PARPi (olaparib). Indicated cells were grown in 96-well plates and treated with different doses of CDEAH with a fixed indicated dose of olaparib for 6 days. Cell viability was determined using Cell Titer-Blue reagent. (**F**) Cell survival in response to different doses of olaparib with CDEAH. Cells were grown in 96-well plates and treated with various doses of olaparib with a fixed indicated dose of CDEAH for 6 days. Cell viability was determined using Cell Titer-Blue reagent. (**G**) CDEAH kills *XPA*- and *CSB*-deficient U2OS cells better than WT. U2OS cells deficient in *XPA*, *XPC*, *CSB* or WT were grown in 96-well plates at a density of 3,000 cells per well and treated with CDEAH for 48 h. Cell viability was determined using Cell Titer-Glo reagent. (**H**) Co-treatment of CDEAH and olaparib shows the effect of CDEAH on lowering the dosage of olaparib to specifically kill BRCA2-deficient cells. IC_50_ was calculated by nonlinear regression (curve fit) using GraphPad Prism (version 9.0.0). Data are presented as mean ± SEM.

Statistical analysis was performed using GraphPad Prism (version 9.0.0). Significance is expressed as *P*-values [*P* > 0.5 (ns), *P* < 0.05 (*), *P* < 0.01 (**), *P* < 0.001 (***), *P* < 0.0001 (****)]. Ordinary one-way ANOVA, Dunnett’s multiple comparison test, with a single pooled variance was used to compare groups (Figure 1B and C). Ordinary two-way ANOVA, Sidak’s multiple comparison test, with a single pooled variance was used to compare groups (Figures 1D–H and 3C–E, G and H). An unpaired, two-tailed *t*-test was used to compare groups (Figure 4C).

## RESULTS

### CDEAH selectively kills XPA- and PARP1-deficient cells

We previously developed a high-throughput genotoxicity screening assay that uses ATAD5 expression as a biomarker to identify genotoxic compounds (33). Using this assay, we identified CDEAH (Figure 1A) that increased the ATAD5-luciferase expression (Figure 1B, PubChem BioAssay AID 720516). Tumors frequently have defects in a DNA repair pathway that renders them vulnerable to certain DNA-damaging agents. For example, *BRCA1*/*2*-deficient tumors, which are unable to perform HR, are sensitive to DNA-damaging agents such as cisplatin or ionizing radiation that require HR for repair. To determine whether any pathway defects render cells sensitive to CDEAH, we incubated HAP1 cell lines with mutations in various DNA repair genes with CDEAH for 48 h and measured cell viability using the Cell Titer-Glo luminescent cell viability assay. Compared to the WT, the *XPA* KO and *PARP1* KO HAP1 cell lines showed significant sensitivity to 20 μM CDEAH treatment (Figure 1C), while mutations in other genes did not show an effect. Similar sensitivity to CDEAH was observed in an HCT116 *PARP1* KO cell line in a dose-dependent manner (Figure 1D). We also measured cell viability in XP2OS cells derived from an *XPA* mutant xeroderma pigmentosum patient and found that they showed significant and dose-dependent sensitivity to CDEAH (Figure 1D). Sensitivity of XP2OS cells to CDEAH was rescued by expression of WT XPA protein complemented with the WT XPA, and the combination of CDEAH and olaparib induced an additive effect in *XPA* KO rather than in WT (Supplementary Figure S2A and B), demonstrating that XPA and PARP1 are both required independently for the sensitivity of CDEAH. The killing effect of CDEAH and olaparib on *XPA* KO cells was analyzed by the CompuSyn program. Based on the analysis, we concluded that CDEAH and olaparib’s effects on *XPA* KO cells were synergistic (Supplementary Figure S2C and D).

To independently confirm the selective sensitivity of *PARP1* KO cell lines to CDEAH, the viability of HAP1 and HCT116 cell lines was evaluated after co-treatment of a fixed concentration of olaparib and an increasing concentration of CDEAH. Co-treatment of olaparib and CDEAH induced hypersensitivity in all tested cell lines in a dose-dependent manner, as we observed *PARP1* KO cell lines (Figure 1E and Supplementary Figure S1C). Co-treatment of a fixed concentration of CDEAH with an increasing dose of olaparib similarly induced hypersensitivity in all HAP1, HCT116 and U2OS cell lines (Figure 1F and Supplementary Figure S1D).

To investigate whether NER is necessary to repair the CDEAH-induced lesions, we conducted a cell viability assay of CDEAH in *XPC* KO and *CSB* KO cell lines. We found that CDEAH induced mild sensitivity in *XPC* KO cells, but severe sensitivity in *CSB* KO cells (Figure 1G), suggesting that CDEAH induces lesions that are dependent more on transcription-coupled NER compared to the global genome NER.

We also tested the effect of CDEAH on lowering the dosage of olaparib to specifically kill *BRCA1*/*2*-deficient cells by performing cell viability assay using TK6 cells with BRCA2-mAID-GFP. We found that incubating this cell with auxin, which degrades BRCA2 protein, resulted in sensitivity to olaparib in a dose-dependent manner. Moreover, co-treatment of CDEAH and olaparib induced hypersensitivity specifically in *BRCA2*-deficient cells (Figure 1H).

Taken together, the result suggests that CDEAH can selectively cause hypersensitivity in cells defective in NER and PARP1-dependent BER pathways.

### Characterization of the reaction of CDEAH with nucleobases

**Figure 2.**
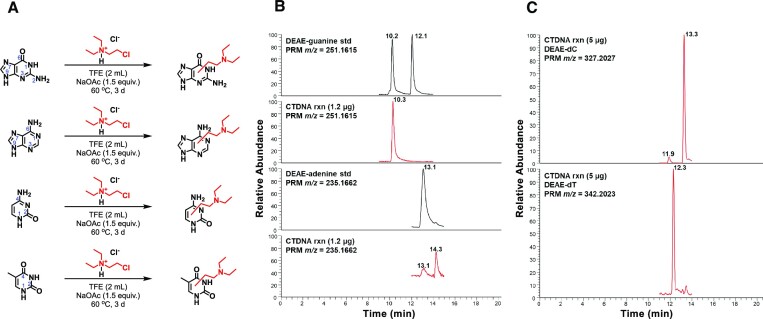
Characterization of alkylated nucleobases by CDEAH. (**A**) Alkylating reactions with different nucleobases by CDEAH. (**B**) Representative UPLC–HRAM-PRM traces of DEAE-purines from CDEAH-treated CTDNA. After incubation with CDEAH for 24 h, alkylated purines were released by thermal hydrolysis and enriched for analysis as described in the ‘Materials and Methods’ section. The black traces represent UPLC–HRAM-PRM analysis of synthesized DEAE-guanine (panel 1, *m*/*z*= 251.1615) and DEAE-adenine (panel 3, *m*/*z*= 235.1662) standards. The red traces represent UPLC–HRAM-PRM of DEAE-guanine (panel 2) and DEAE-adenine (panel 4) detected from 1.2 μg depurinated CTDNA. (**C**) Representative UPLC–HRAM-PRM traces of DEAE-pyrimidines enzymatically released from 5 μg CDEAH-treated CTDNA. The top panel represents DEAE-dC (*m*/*z*= 327.2027) and the bottom panel represents DEAE-dT (*m*/*z*= 342.2023).

CDEAH is half-nitrogen mustard, and its Cl group can be displaced by an intramolecular ring-closing reaction to yield a highly electrophilic aziridinium ion, which can alkylate the nucleophilic positions of DNA nucleobases. To test this potential mechanism, we investigated the alkylation of purine and pyrimidine nucleobases by CDEAH by incubating 0.2 mmol of each nucleobase with 0.2 mmol of CDEAH (Figure 2A) (38). Reaction mixtures were purified by SPE and analyzed by UPLC–HRAM-PRM. Using this assay, the desired DEAE-adenine (*m*/*z* = 235.1671; Scheme 1), DEAE-guanine (*m*/*z* = 251.1611; Scheme 2), DEAE-thymine (*m*/*z* = 226.0972; Scheme 3) and DEAE-cytosine (*m*/*z* = 211.1551; Scheme 4) were identified eluting at 13.1 and 14.3 (weak peak), 10.2 and 12.1, 11.0 and 11.5, and 4.8 and 9.2 min, respectively (Supplementary Figure S3A). PRM fragmentation revealed that all four DEAE-nucleobases shared the same major fragment of cleavage at the diethyl linker to yield *N*,*N*-diethylethenaminium [M − base + H]^+^, *m*/*z* = 100.1124 and an *N*-vinyl nucleobase ion [M − NC_4_H_11_]^+^; DEAE-adenine *m*/*z* = 235.1671 to 162.0772, DEAE-guanine *m*/*z* = 251.1611 to 178.0721, DEAE-thymine *m*/*z* = 226.0972 to 153.0656 and DEAE-cytosine *m*/*z* = 211.1551 to 138.0661 (Supplementary Figure S3A). Analogous reactions with 2-bromo-*N*,*N*-diethyethan-1-amine yielded identical products but at very reduced yields, with the acetate-quenched 2-(diethylamino)ethyl acetate as the major product. Based on the UPLC–HRAM-PRM results, the observation of multiple peaks with similar fragmentation patterns would indicate that cross-linking likely occurred at the N1, N3, N7, N9 or *O*^6^ positions of guanine, the N1, N3, N7 or N9 positions of adenine, the N1, *O*^2^ or N3 positions of cytosine, and the N1, *O*^2^, N3 or *O*^4^ positions of thymine, while the connectivity of the DEAE to the base was not revealed by our studies.

### Confirmation and characterization of alkylated CTDNA by CDEAH

To further investigate CDEAH alkylation on nucleotides in double-stranded DNA, CTDNA was incubated with CDEAH for 16 h. Alkylated purines were released from the DNA backbone by thermal hydrolysis and analyzed by the UPLC–HRAM-PRM assays (39,40). When the equivalent of 240 ng of CTDNA was analyzed, we were able to confirm the presence of DEAE-guanine at 10.3 min (Figure 2B). Since the N9 position of guanine is not accessible in double-stranded DNA, the observed peak at 10.3 min is expected to be N7-DEAE-guanine and the standard peak at 12.1 min is expected to be N9-DEAE-guanine. During the analysis of 240 ng of CTDNA, we were unable to detect any DEAE-adenine. When the scale of the reaction was increased 5-fold to 1.2 μg of CTDNA, we were able to detect a weak signal for DEAE-adenine at both 13.1 and 14.3 min (Figure 2B). Analysis of the PRM data revealed that both peaks had the expected fragmentation pattern. Given that the N3 position of 2′-deoxyadenosine is the most reactive position with other nitrogen mustards in DNA (38,41), we believe the weak signal at 13.1 min corresponds to the N7-DEAE-adenine and the stronger signal at 14.3 min corresponds to N3-DEAE-adenine.

The remaining alkylated DNA backbone was digested with NEB nucleoside digestion mix to yield DEAE-2′-deoxypyrimidines. When 5 μg of CTDNA digestion was analyzed by the modified DEAE-pyrimidine nucleoside UPLC–HRAM-PRM assay, we were able to detect two DEAE-dC peaks at 11.9 and 13.3 min and one DEAE-dT peak at 12.3 min (Figure 2C). The DEAE-dC peak at 11.9 min yielded fragments of *m*/*z* = 211.1511, 138.0661 and 100.1124 corresponding to cleavage of the 2′-deoxysugar [M − dR + H]^+^ and at the diethyl linker to yield *N*-vinyl-cytosine ions [M − NC_4_H_11_ − dR + H]^+^ and *N*,*N*-diethylethenaminium [M − dC + H]^+^ (Supplementary Figure S3B). The DEAE-dC peak at 13.3 min yielded fragments of *m*/*z* = 283.17488, 177.11195, 133.08587 and 89.06019, which could not be identified. The DEAE-dT peak at 12.3 min yielded fragments of *m*/*z* = 226.1547, 153.0656 and 100.1124 corresponding to cleavage of the 2′-deoxysugar [M − dR + H]^+^ and at the diethyl linker to yield *N*-vinyl-cytosine ions [M − NC_4_H_11_ − dR + H]^+^ and *N*,*N*-diethylethenaminium [M − dT + H]^+^ (Supplementary Figure S3B). Given that the N1 position of pyrimidines is not accessible in nucleotides, the observed peak at 11.9 min is expected to correspond to *O*^2^-DEAE-dC and the peak at 12.1 min is expected to correspond to *O*^2^-DEAE-dT or *O*^4^-DEAE-dT. Taken together, CDEAH is most reactive to 2′-deoxyguanosine, followed by 2′-deoxyadenosine and then 2′-deoxypyrimidines (${\rm dG}\gg {\rm dA} >{\rm dC} \sim {\rm dT}$).

To investigate reactivity and alkylation potential, we treated WT and mutant HAP1 cell lines with a CDEAH derivative that replaced the chloride-leaving group with a bromide-leaving group (Supplementary Figure S1A and B). However, there was no significant increase in sensitivity with the bromide-substituted derivative compared to CDEAH.

### CDEAH induces more DNA breaks in PARP1-deficient cells

**Figure 3.**
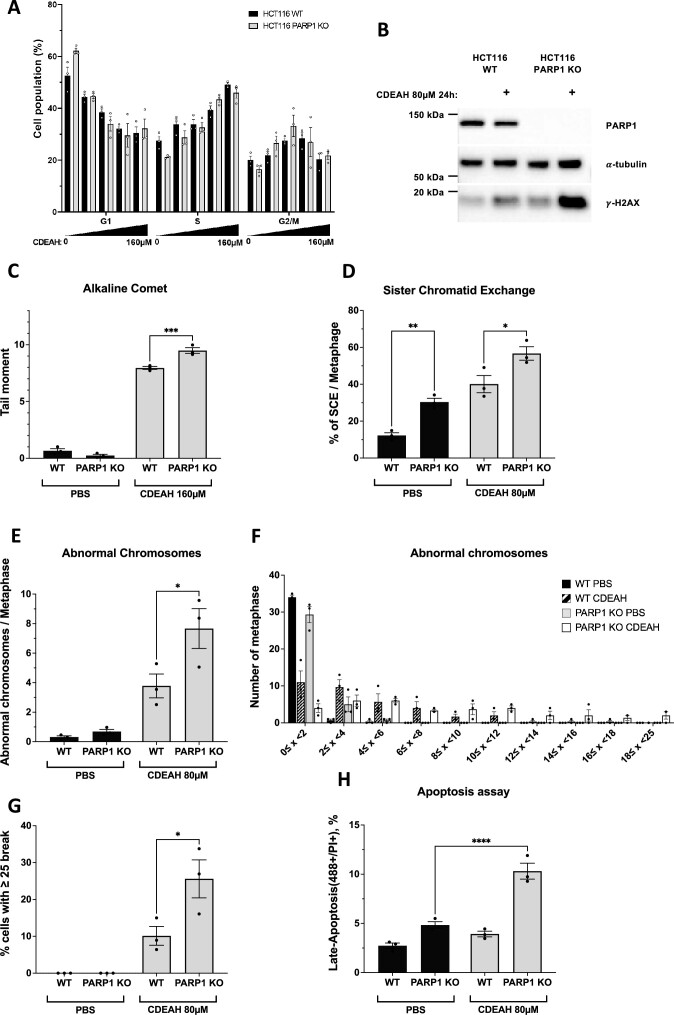
CDEAH induces more DNA double-strand breaks (DSBs) in PARP1-deficient cells. (**A**) Cell cycle of HCT116 after CDEAH treatment. HCT116 WT and PARP1-deficient cells were incubated with different doses of CDEAH for 24 h and the relative percentage of cell cycle stages was calculated by FlowJo software. (**B**) DSB occurrence caused by CDEAH treatment was confirmed by γ-H2AX. HCT116 WT or PARP1-deficient cells were incubated with 80 μM CDEAH for 24 h and indicated protein level was determined in whole-cell extracts. (**C**) CDEAH treatment enhances DNA damage in HCT116 PARP1-deficient cells. The tail moment in the CometChip^®^ assay was calculated using the Comet analysis software (Trevigen). (**D**) SCE analysis in HCT116 WT and PARP1-deficient cells. SCEs were imaged by a BX53 microscope. At least 20 metaphases per each condition were analyzed. (**E**) Abnormal chromosomes were analyzed in HCT116 WT and PARP1-deficient cells. Abnormal chromosomes were imaged by a BX53 microscope. At least 20 metaphases per each condition were analyzed. (**F**) Graph displaying the number of SCEs in one metaphase in different conditions. (**G**) Cells that have >25 breaks in one metaphase were analyzed from panel (E). (**H**) CDEAH treatment causes more apoptotic cell death in PARP1-deficient cells. Apoptotic cell death was quantified using an Annexin V Alexa Fluor™ 488 conjugate and analyzed by flow cytometry. Data are presented as mean ± SEM.

Since CDEAH alkylates nucleobases, CDEAH treatment is expected to interfere with replication and S-phase progression. We investigated the effect of CDEAH on cell cycle progression in HCT116 WT and *PARP1* KO cell lines. Both WT and *PARP1* KO cells were arrested at S phase upon treatment with increasing concentrations of CDEAH, but there was no significant difference in WT and *PARP1* KO cells (Figure 3A). We confirmed that CDEAH also increases PAR, as previous studies have reported (42,43) an increase in PAR levels by alkylating agents (Supplementary Figure S5A). Since *PARP1* KO cells were selectively killed by CDEAH, we compared DNA damage markers after 24-h treatment of 80 μM CDEAH in WT and *PARP1* KO cells. Consistent with cell viability results, we found higher γ-H2AX induction in CDEAH-treated *PARP1* KO cells (Figure 3B). Furthermore, actual single-stranded DNA breaks measured by the alkaline comet assay were increased in *PARP1* KO cells compared to WT cells upon CDEAH treatment (Figure 3C). To investigate genomic instability, we tested sister-chromatid exchange (SCE) frequency and abnormal chromosomes in HCT116 WT and *PARP1* KO cells after 24-h treatment with 80 μM CDEAH. Consistent with a previous study, *PARP1*-deficient cells showed increased SCE frequency even in the absence of exogenous damage (44–47). We found a further increase in SCE frequency and abnormal chromosomes in CDEAH-treated *PARP1* KO cells (Figure 3D–F). For example, cells with >25 breaks per metaphase were significantly increased in CDEAH-treated *PARP1* KO cells (Figure 3G). Finally, apoptotic cell death was quantified using an Annexin V Alexa Fluor™ 488 conjugate. Compared to the WT, the *PARP1* KO cell line showed significantly increased apoptosis upon treatment with 80 μM CDEAH (Figure 3H). These results show that DNA damage, chromosomal aberrations and apoptotic cell death are increased in *PARP1*-deficient cells following treatment with CDEAH.

### Growth of *PARP1* KO xenograft tumors is selectively inhibited by CDEAH treatment

**Figure 4.**
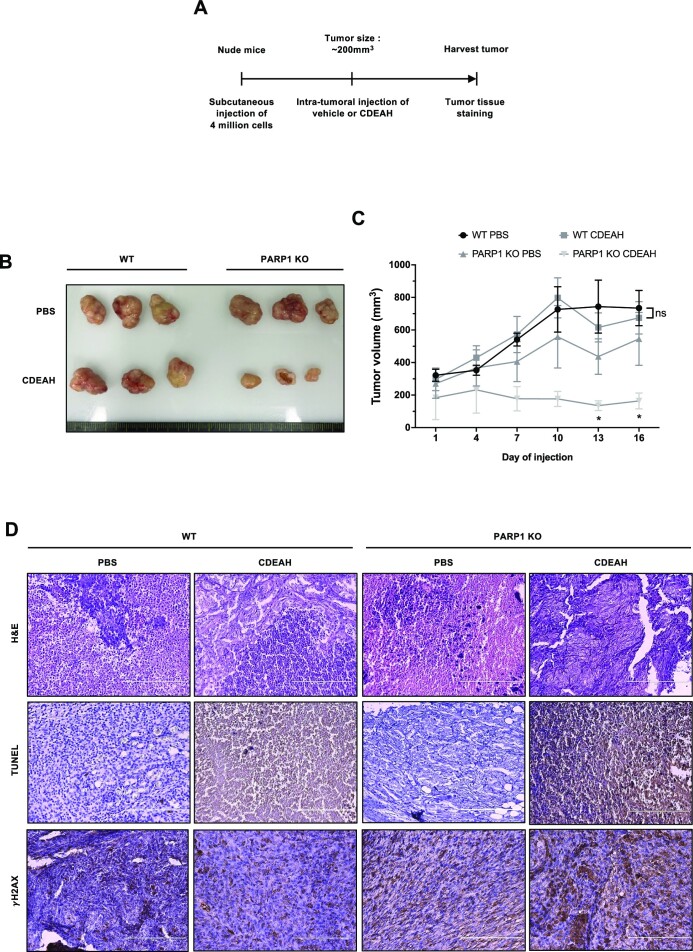
CDEAH inhibits the growth of PARP1-deficient xenograft tumors in nude mice. (**A**) Scheme of *in vivo* xenograft experiment. Four million cells of either WT HCT116 or PARP1-deficient HCT116 cells were subcutaneously injected into seven-week-old male nude mice. When the tumor size reached ∼200 mm^3^, vehicle (PBS) or CDEAH (6 mg/kg) was injected intratumorally every 3 days for 16 days. The mice were euthanized, followed by the indicated analyses. (**B**) Representative orthotopic xenografts of each indicated group (*n* = 3). (**C**) Tumor volume change was measured every 3 days for 16 days during drug treatment [*n* = 5, WT/PBS (black circle); 5, WT/CDEAH (gray square); 4, PARP1-deficient/PBS (gray triangle); 6, PARP1-deficient/CDEAH (pale gray inverted triangle)]. (**D**) Representative images of H&E, TUNEL and γ-H2AX staining of each dissected xenograft tumor. Data are presented as mean ± SEM.

To determine the effect of CDEAH on tumor growth *in vivo*, we analyzed the susceptibility of tumor xenografts in nude mice to treatment with CDEAH (Figure 4A). Four million WT or *PARP1* KO HCT116 cells were subcutaneously injected into the left flanks to form xenografted tumors. When tumor size reached ∼200 mm^3^, vehicle or CDEAH was injected intratumorally. Vehicle-treated WT and *PARP1* KO tumors grew continuously. In contrast to the continual growth of WT tumors, HCT116 *PARP1* KO tumors showed significant retardation of growth following CDEAH treatment (Figure 4B). The regularly traced volume of xenograft tumors of each group demonstrated that CDEAH selectively retarded the proliferation of *PARP1*-deficient HCT116 xenografted tumors (Figure 4C). Histology of apoptotic cell death and DNA damage was investigated by the TUNEL assay and γ-H2AX immunostaining (Figure 4D). Consistent with *in vitro* data, CDEAH treatment caused apoptotic cell death and induced γ-H2AX formation. Altogether, our data show that CDEAH specifically restrains the growth of PARP1-deficient tumors *in vivo*.

## DISCUSSION

In the present study, we identified CDEAH as an agent that selectively kills PARP1- and XPA-deficient cells *in vitro* and *in vivo*. Our data suggest that co-treatment of PARPi such as olaparib with CDEAH enhances tumor growth inhibition. CDEAH preferentially alkylates guanine DNA bases, which subsequently interferes with progression through S phase, which is further enhanced in PARP1-deficient cells leading to cell death. The PARP1 dependence of CDEAH covalent adducts gives a potential synergistic combination treatment option with PARPi. PARPi such as olaparib, rucaparib, niraparib, talazoparib and veliparib are FDA approved for clinical usage (17). In addition to monotherapy of these PARPi, various combinatorial treatments with PARPi are currently being investigated in clinical trials. For example, TMZ has been used in combinatorial therapy with rucaparib ever since the first clinical trial of a PARPi in 2003 (17). Increased sensitivity of cells with reduced PARP1 activity to the TOP1 inhibitor camptothecin, irinotecan and topotecan, which is used as a cancer therapeutic agent, raised a potential combinatorial treatment of TOP1i with PARPi (11,17,48–52). In addition, various combinatorial therapies are currently undergoing clinical trials for PARPi in various cancers (11,17).

There are many other well-characterized DNA alkylating agents, such as nitrogen mustard compounds, cisplatin and MMS (53–57). Nitrogen mustard compounds and cisplatin have two electrophiles (Cl ligands) and form an inter- or intrastrand cross-link between two nucleobases in addition to monoadducts. Such complex adducts induce various higher toxic effects on both tumor and normal cells that need to be overcome by either the NER or inter-cross-link repair pathway for cell survival (55–61). In contrast to these agents, since CDEAH possesses a single electrophile, it makes only one type of DNA adduct, monoadduct. These simple adducts are substrates for either the BER or NER pathways, and thus may induce milder side effects on normal cells. The alkylating agent MMS makes a simple monoadduct with DNA nucleobases similar to CDEAH (53,54). Previous experiments reported a synergistic effect of 0.01% MMS with olaparib (62,63), whereas XPA deficiency did not show an effect (64,65). To confirm these, we incubated HAP1 cell lines with PARP1 or XPA deficiency with MMS or TMZ for 48 h and measured cell viability. Our results showed that only the PARP1 KO HAP1 cell lines exhibited significant sensitivity to MMS, while both PARP1 KO and XPA KO HAP1 cell lines had minor effects with TMZ (Supplementary Figure S4A and B). In contrast, the CDEAH shows synergistic effect with olaparib and selectively kills not only PARP1- but also XPA-deficient cells (Figure 1). A previous study reported that monoadducts of melphalan with two electrophiles (Cl ligands) can be repaired by NER in human cell-free extracts (66). Taken together, CDEAH covalent adducts are bulky and subject to NER. Lastly, the high water solubility of CDEAH compared to other cross-linking reagents is another beneficial characteristic of CDEAH for combinatorial treatment.

CDEAH sensitizes cells defective in either NER or PARP1-dependent BER pathways. Thus, in addition to the potential clinical uses of CDEAH for cancer therapy, CDEAH can be used as a tool compound to better understand NER and PARP1-dependent BER pathways in detail. When CDEAH alkylates the DNA nucleobase, a very strong covalent bond is formed between CDEAH and the nucleobase, especially guanine. CDEAH adducts should be removed from the genome by the NER or PARP1-dependent BER pathway. In combination with the UPLC–HRAM-PRM assay used in this study, the removal of CDEAH adducts from the genome can be analyzed to study the kinetics of the NER and PARP1-dependent BER pathways in various DNA repair-deficient cell lines as well as cancer cells. Since remaining CDEAH adducts would cause mutations in the genome, CDEAH could also be used to study mutagenesis mechanisms in cells. CDEAH preferentially alkylates guanine bases, suggesting that a unique mutation signature would be produced in cells defective in a different DNA repair pathway. The analysis of mutation signatures accumulated in CDEAH-treated cancer cells with different genetic backgrounds could be used as references to choose treatment options.

CDEAH treatment increased the population of S-phase cells. This could be due to the stalling of DNA replication by the CDEAH-alkylated nucleobases. DNA damage in the genome at the S phase is recognized by the MMR machinery or bypassed by the translesion synthesis (TLS) pathway (67). It would be interesting to investigate whether CDEAH adducts at guanine induce effects on the MMR pathway. CDEAH adducts encountering DNA replication forks would be bypassed by the TLS pathway. Since TLS is an error-prone pathway that generates mutations in the genome, it will be interesting to investigate what type of mutations would be produced by CDEAH and which TLS enzymes are involved in mutagenesis. In addition to DNA replication, CDEAH adducts in the coding sequences could affect transcription. The preferential alkylation of guanine bases suggests that CDEAH would target CpG islands as a transcriptional obstacle. Most CpG islands in promoter are unmethylated, but in the silenced promoter, CpG islands are usually methylated and are important for the silencing state of the promoter (68–70). CDEAH could impact this regulation as well. It will be interesting to investigate the transcriptional impact of CDEAH on genes carrying CpG island in their promoters. Other sequence-dependent DNA structures in the genome, such as G-quadruplex-rich sequences frequently found in telomeres (71,72), could be affected by CDEAH as well.

Collectively, we identified a small molecule, CDEAH, as a potential sensitizing agent for PARPi and a tool compound to study various DNA repair pathways. The development of sensitizers using the synthetic lethality strategy used in this study suggests that many other small molecules can be developed to target various genetic diseases, including cancers.

## Supplementary Material

zcad042_Supplemental_Files

## Data Availability

The data underlying this article are available in the article and in its online supplementary material.
