# Supplementary material for: Alkylation of nucleobases by 2-chloro-N,N-diethylethanamine hydrochloride (CDEAH) sensitizes PARP1-deficient tumors
Source: NAR Cancer. 2023 Aug 7;5(3):zcad042. doi: 10.1093/narcan/zcad042 (PMC10405566; doi:10.1093/narcan/zcad042)

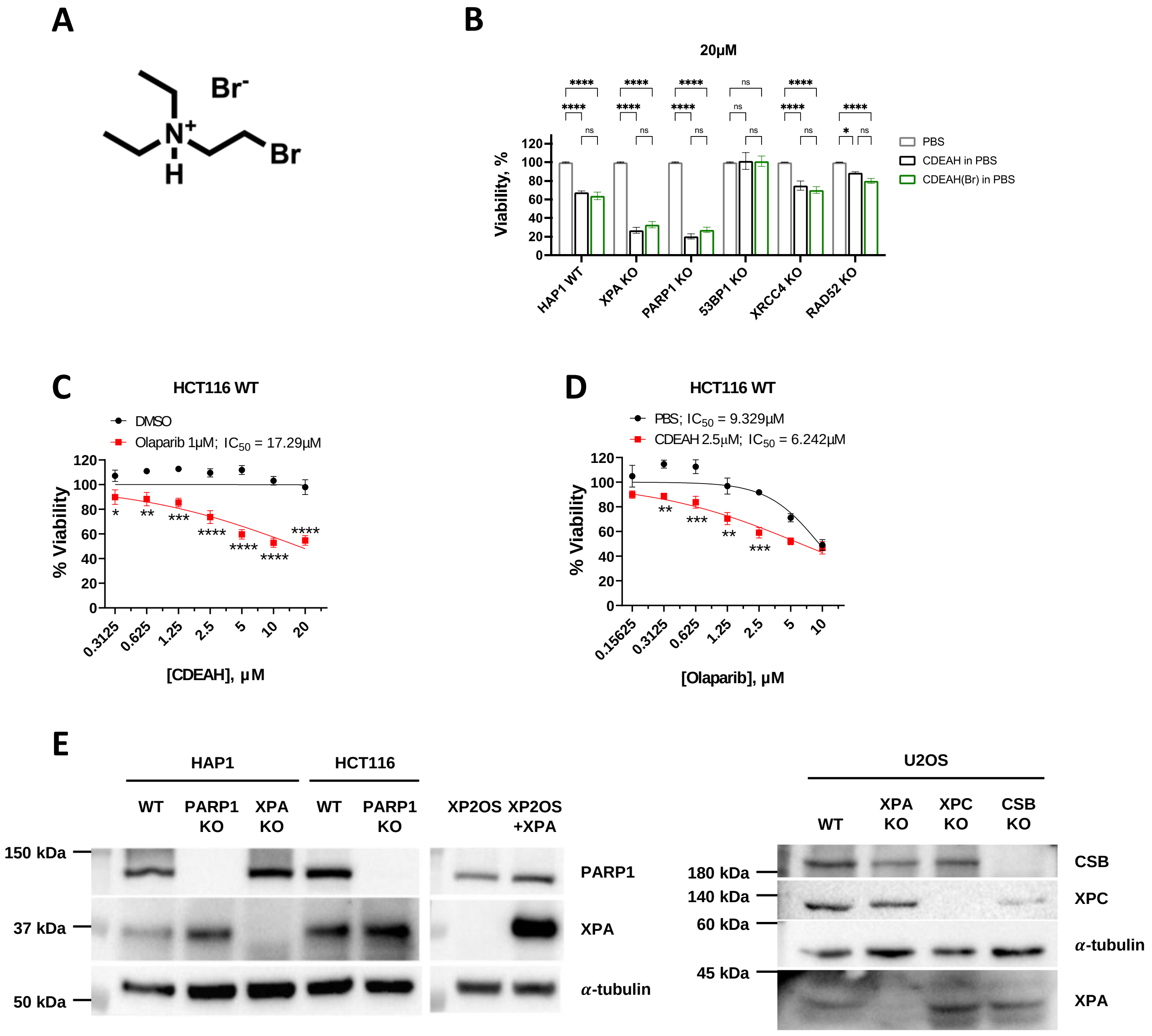

Wie et al., Sup. Figure 1

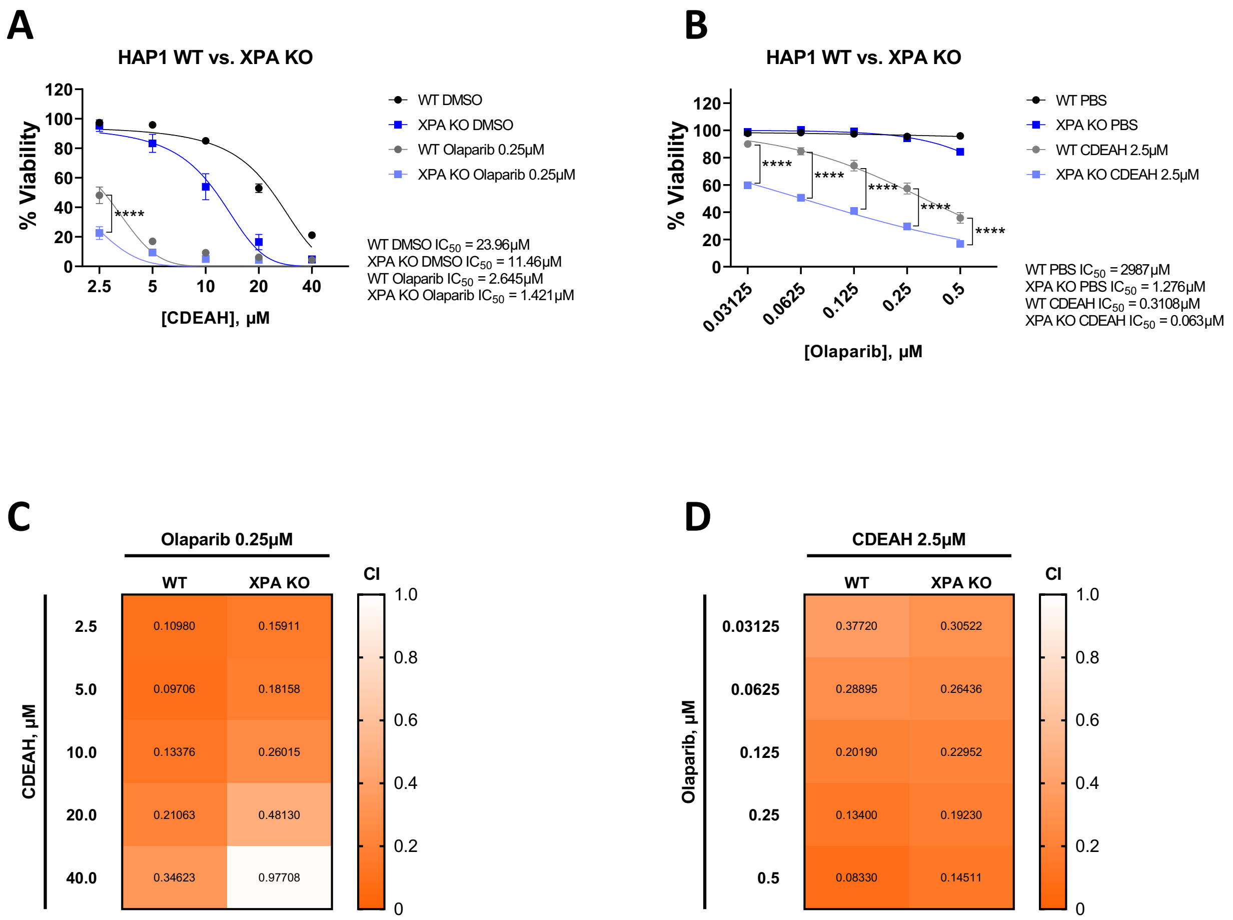

C — Olaparib 0.25μM

CDEAH, μM	WT	XPA KO
2.5	0.10980	0.15911
5.0	0.09706	0.18158
10.0	0.13376	0.26015
20.0	0.21063	0.48130
40.0	0.34623	0.97708

D — CDEAH 2.5μM

Olaparib, μM	WT	XPA KO
0.03125	0.37720	0.30522
0.0625	0.28895	0.26436
0.125	0.20190	0.22952
0.25	0.13400	0.19230
0.5	0.08330	0.14511

A

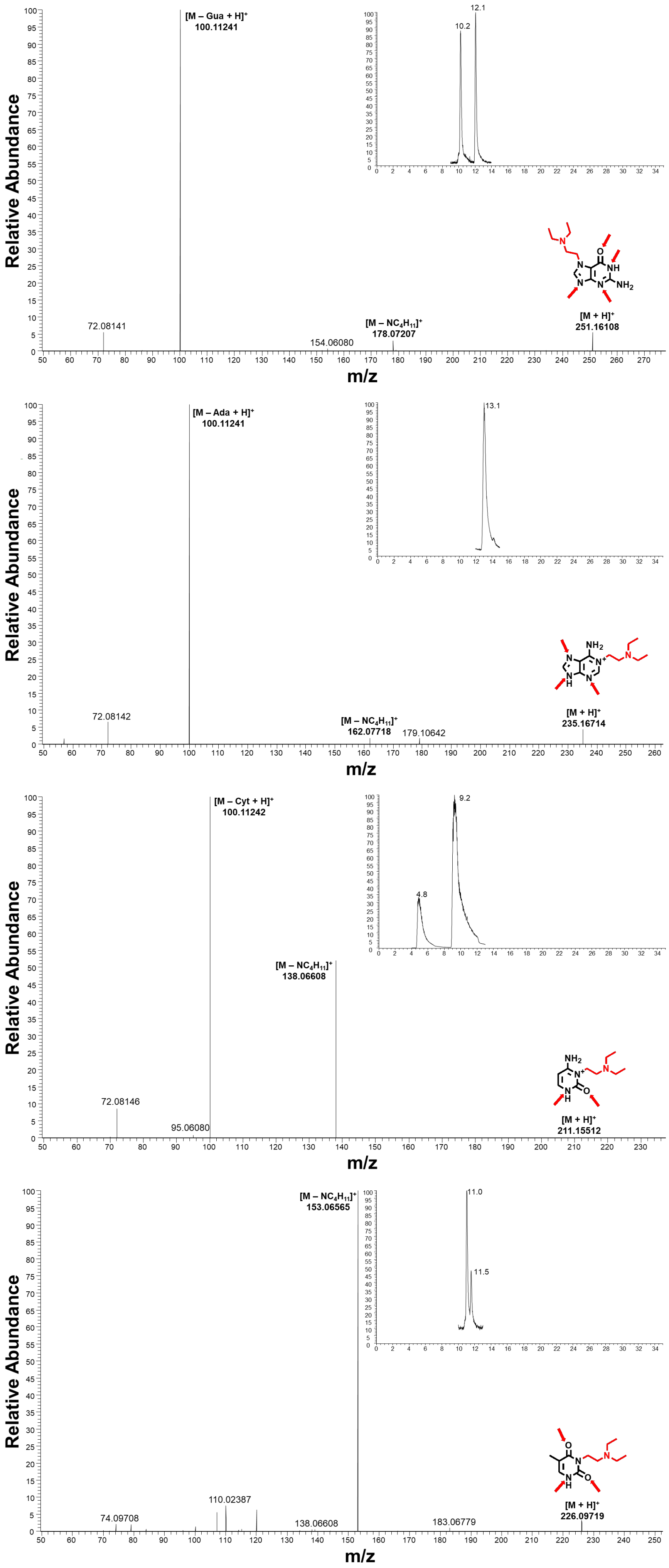
Relative Abundance
m/z
[M – Gua + H]+ 100.11241
72.08141
154.06080
[M – NC4H11]+ 178.07207
[M + H]+ 251.16108
10.2
12.1
[M – Ada + H]+ 100.11241
72.08142
[M – NC4H11]+ 162.07718
179.10642
[M + H]+ 235.16714
13.1
[M – Cyt + H]+ 100.11242
[M – NC4H11]+ 138.06608
72.08146
95.06080
[M + H]+ 211.15512
4.8
9.2
[M – NC4H11]+ 153.06565
74.09708
110.02387
138.06608
183.06779
[M + H]+ 226.09719
11.0
11.5

B

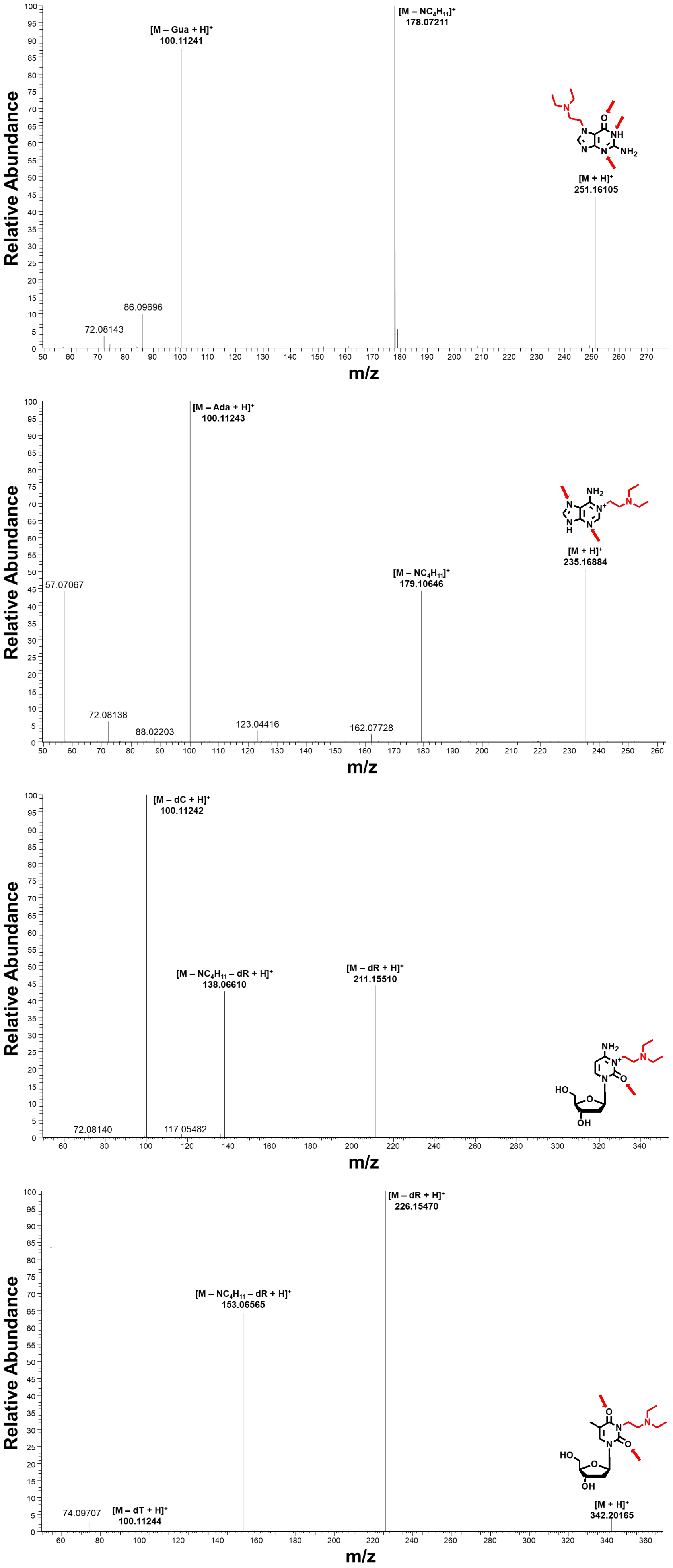

Relative Abundance
m/z
[M – Gua + H]+
100.11241
[M – NC4H11]+
178.07211
[M + H]+
251.16105
86.09696
72.08143
[M – Ada + H]+
100.11243
[M + H]+
235.16884
[M – NC4H11]+
179.10646
57.07067
72.08138
88.02203
123.04416
162.07728
[M – dC + H]+
100.11242
[M – NC4H11 – dR + H]+
138.06610
[M – dR + H]+
211.15510
72.08140
117.05482
[M – dR + H]+
226.15470
[M – NC4H11 – dR + H]+
153.06565
74.09707
[M – dT + H]+
100.11244
[M + H]+
342.20165

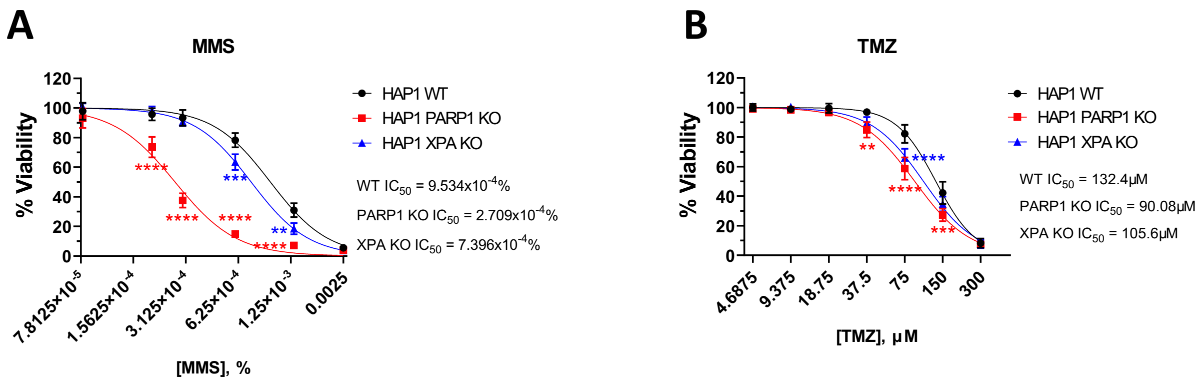

Wie et al., Sup. Figure 4

A

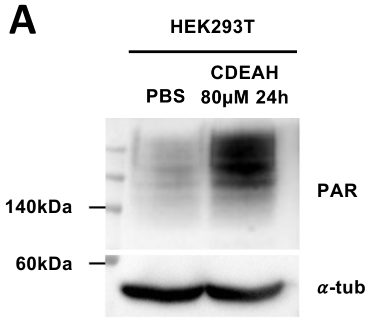

A

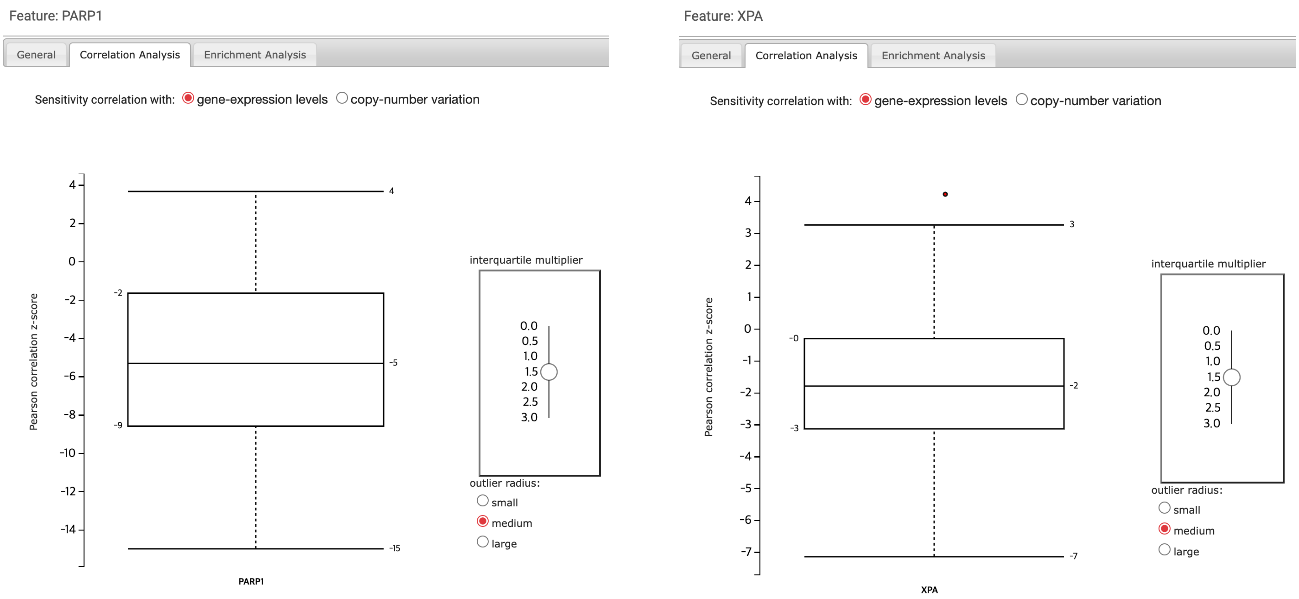

B

Mutation distribution

This section displays a series of charts that show the distribution of different types of mutations for PARP1.

Summary

An overview of the types of mutation observed.

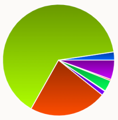

Colour	Mutation type	Number of samples (%)
	Nonsense substitution	18 (1.85%)
	Missense substitution	492 (50.57%)
	Synonymous substitution	171 (17.57%)
	Inframe insertion	0 (0.00%)
	Frameshift insertion	10 (1.03%)
	Inframe deletion	1 (0.10%)
	Frameshift deletion	25 (2.57%)
	Complex mutation	3 (0.31%)
	Other	43 (4.42%)
	Total unique samples	973

Mutation distribution

This section displays a series of charts that show the distribution of different types of mutations for XPA.

Summary

An overview of the types of mutation observed.

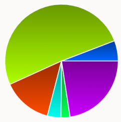

Colour	Mutation type	Number of samples (%)
	Nonsense substitution	9 (3.72%)
	Missense substitution	79 (32.64%)
	Synonymous substitution	22 (9.09%)
	Inframe insertion	0 (0.00%)
	Frameshift insertion	0 (0.00%)
	Inframe deletion	6 (2.48%)
	Frameshift deletion	4 (1.65%)
	Complex mutation	0 (0.00%)
	Other	35 (14.46%)
	Total unique samples	242

Original Data

Figure 3B

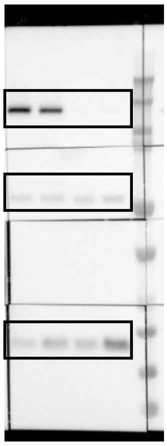

Supplementary Figure 1E

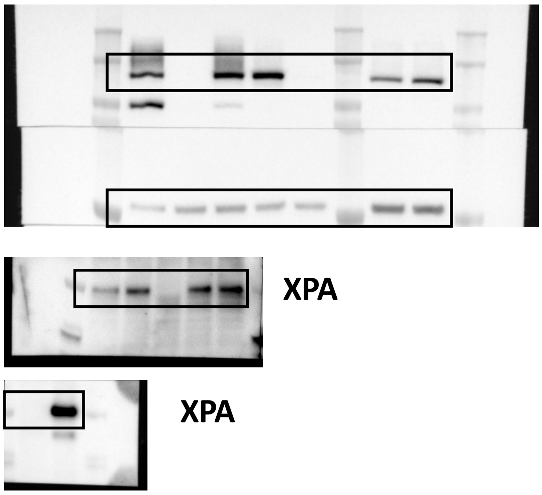

Supplementary Figure 1E

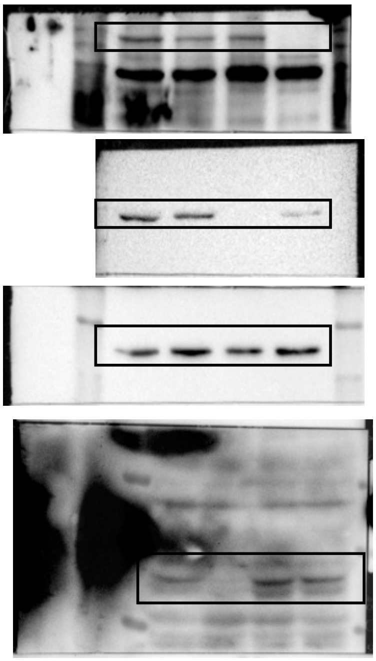

Supplementary Figure 5A

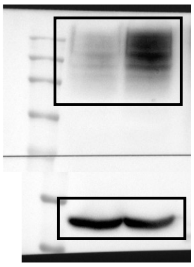

Supplement: zcad042_Supplemental_Files [file zcad042_supplemental_files.zip › Wie et al supplement figures.pdf]
