# Supplementary material for: Alkylation of nucleobases by 2-chloro-N,N-diethylethanamine hydrochloride (CDEAH) sensitizes PARP1-deficient tumors
Source: NAR Cancer. 2023 Aug 7;5(3):zcad042. doi: 10.1093/narcan/zcad042 (PMC10405566; doi:10.1093/narcan/zcad042)
Supplement: zcad042_Supplemental_Files [file zcad042_supplemental_files.zip › Wie et al supplementary text.docx]

**Alkylation of nucleobases by 2-chloro-*N,N*-diethylethanamine hydrochloride (CDEAH) sensitizes *PARP1*-deficient tumors**

Supplementary text

By

Minwoo Wie^1,2^*, Keon Woo Khim^1,2^*, Arnold S. Groehler IV^1^, Soomin Heo^1,4^, Junhyeok Woo^3^, Kook Son^1^, Eun A Lee^1^, Jae Sun Ra^1^, Sung You Hong^3^, Orlando D. Schärer^1,2+^, Jang Hyun Choi^1,2+^, and Kyungjae Myung^1,4+^

1. Center for Genomic Integrity, Institute for Basic Science, Ulsan 44919, Republic of Korea.

2. Department of Biological Sciences, Ulsan National Institute of Science and Technology, Ulsan 44919, Republic of Korea

3. Department of Chemistry, Ulsan National Institute of Science and Technology, Ulsan 44919, Republic of Korea

4. Department of Biomedical Engineering, Ulsan National Institute of Science and Technology, Ulsan 44919, Republic of Korea

*Equal contributions

+corresponding authors

Email: [janghchoi@unist.ac.kr](mailto:janghchoi@unist.ac.kr) (Jang Hyun Choi), [orlando.scharer@ibs.re.kr](mailto:orlando.scharer@ibs.re.kr) (Orlando Schärer, [kmyung@ibs.re.kr](mailto:kmyung@ibs.re.kr) (Kyungjae Myung)

**Supplementary Figure 1. Cell viability of CDEAH or Br-substituted derivative in DNA repair gene KO cells.** (**A**) Structure of bromide-substituted derivative. (**B**) Cell viability in various DNA repair gene KO HAP1 cells with CDEAH and bromide-substituted derivative. Wild-type (WT) or various DNA repair gene KO HAP1 cells were grown in 96-well plates at a density of 5,000 cells per well and treated with 20 µM CDEAH or bromide-substituted derivative for 48 hours. Cell viability was determined using Cell Titer-Glo reagent. (**C**) Cell survival in response to various doses of CDEAH with PARP inhibitor (Olaparib). HCT116 WT cells were grown in 96-well plates and treated with different doses of CDEAH with a fixed indicated dose of Olaparib for 6 days. Cell viability was determined using Cell Titer-Blue reagent. (**D**) Cell survival in response to various doses of Olaparib with CDEAH. HCT116 WT cells were grown in 96-well plates and treated with different doses of Olaparib with a fixed indicated dose of CDEAH for 6 days. Cell viability was determined using Cell Titer-Blue reagent. IC_50_ was calculated by nonlinear regression (curve fit) using GraphPad Prism (Version 9.0.0). (**E**) Representative images of western blot from each cell line of Figure 1. 𝛼-tubulin was used as a loading control.

**Supplementary Figure 2. Co-treatment of CDEAH and Olaparib induced hypersensitivity in *XPA* KO than in WT.** (**A**) Cell survival in response to various doses of CDEAH with PARP inhibitor (Olaparib) in HAP1 WT or *XPA* KO cell lines. Indicated cells were grown in 96-well plates and treated with different doses of CDEAH with a fixed indicated dose of Olaparib for 48 hours. Cell viability was determined using Cell Titer-Glo reagent. (**B**) Cell survival in response to different doses of Olaparib with CDEAH in HAP1 WT or *XPA* KO cell lines. Cells were grown in 96-well plates and treated with various doses of Olaparib with a fixed indicated dose of CDEAH for 48 hours. (**C**-**D**) the drug combination matrix for assessing the synergism of indicated concentrations of CDEAH and Olaparib in panels (**A**) and (**B**) using the combination index (CI) method, where values of CI<1 indicate synergism, CI=1 suggest an additive effect, and CI>1 corresponds to antagonism effects. These analyses were performed using CompuSyn software tool. Cell viability was determined using Cell Titer-Glo reagent. IC_50_ was calculated by nonlinear regression (curve fit) using GraphPad Prism (Version 9.0.0). Data are presented as mean ± SEM.

**Supplementary Figure 3. Characterization of synthesized standards.** (**A**) Representative ESI^+^-PRM product ion spectrums for synthesized standards of DEAE-guanine (panel 1, parent *m/z* = 251.1611, DEAE-adenine (panel 2, parent *m/z* = 235.1671), DEAE-cytosine (panel 3, parent *m/z* = 211.15512), and DEAE-thymidine (panel 4, parent *m/z* = 226.0972). The red arrows represent other potential DEAE alkylation positions on the nucleobases. (**B**) Representative ESI^+^-PRM product ion spectrums corresponding to Figure 2B (DEAE-purines) and Figure 2C (DEAE-pyrimidines). Panel 1 is the product ion spectrum for DEAE-guanine (10.3 min, *m/z* = 251.1611). Panel 2 is the product ion spectrum for DEAE-adenine (14.3 min, *m/z* = 235.1688) and the same spectrum was observed at 13.1 minutes. Panel 3 is the product ion spectrum for DEAE-dC (11.9 min, *m/z* = 327.2027), but a different spectrum was observed at 13.3 minutes. This product could not be characterized by the UPLC-HRAM-PRM analysis. Panel 4 is the product ion spectrum for DEAE-dT (12.3 min, *m/z* = 342.2017). The red arrows represent other potential DEAE alkylation positions on the nucleobases or nucleosides.

**Supplementary Figure 4.** **Cell viability of other alkylating agents in DNA repair gene KO cells.**

(**A**) Cell survival in response to various doses of MMS in HAP1 WT, *PARP1* KO, or *XPA* KO cell lines. Indicated cells were grown in 96-well plates and treated with different doses of MMS for 48 hours. Cell viability was determined using Cell Titer-Glo reagent. (**B**) Cell survival in response to various doses of TMZ in HAP1 WT, *PARP1* KO, or *XPA* KO cell lines. Indicated cells were grown in 96-well plates and treated with different doses of TMZ for 48 hours. Cell viability was determined using Cell Titer-Glo reagent. IC_50_ was calculated by nonlinear regression (curve fit) using GraphPad Prism (Version 9.0.0).

**Supplementary Figure 5.** **CDEAH induces PARylation.**

(**A**) PARylation caused by CDEAH treatment was confirmed. HEK293T cells were incubated with 80 µM CDEAH for 24 hours and indicated protein level was determined in whole-cell extracts.

**Supplementary Figure 6. Expression and mutation analysis of PARP1 and XPA in CTRP and Catalogue of Somatic Mutations in Cancer (COSMIC).**

(**A**) Gene-expression levels (any primary site/subtype, any growth mode) of PARP1 and XPA in Cancer Therapeutics Response Portal (CTRP). (**B**) Mutation distributions of PARP1 and XPA in Catalogue of Somatic Mutations in Cancer (COSMIC).
